# A phenomenological exploration of the feedback experience of medical students after summative exam failure

**DOI:** 10.1186/s12909-023-04892-z

**Published:** 2023-12-08

**Authors:** Robert Jay, Pamela Hagan, Christopher Madan, Rakesh Patel

**Affiliations:** 1 University of Lincoln, Lincoln, UK; 2 Edge Hill University, Ormskirk, UK; 3 University of Nottingham, Nottingham, UK; 4 Barts and The London School of Medicine, Queen Mary University of London, London, UK

**Keywords:** Emotion, Feedback, Remediation, Summative exam failure, Undergraduate medicine

## Abstract

**Background:**

Preventing medical students entering cycles of underperformance following assessment is a priority due to the consequences for the student, faculty, and wider society. The benefits from feedback may be inadequately accessed by students in difficulty due to the emotional response evoked by examination failure. This study aims to explore medical students’ experiences of receiving feedback after summative assessment failure and investigate the role of emotions on motivation for learning after underperformance, to better support remediation and preparation for future assessments.

**Methods:**

This study used interpretative phenomenological analysis (IPA) to explore the experiences of four medical students who failed summative assessments. Additionally, a content analysis was conducted using Linguistic Inquiry and Word Count (LIWC) to investigate the characteristics and use of language to describe their emotional response.

**Results:**

Anger, fear, anxiety, and sadness were emotions frequently experienced after examination failure. These emotions led to feelings of mistrust of the medical school and subsequent distrust in the university’s assessment processes, impacting on the desire to engage with feedback. There was dissonance between the students' perceptions of what feedback should provide and what benefit feedback provided after summative assessments. The linguistic inquiry further confirmed an initial (and sometimes long lived) negative affective state after experiencing failure, and a barrier to engagement with remediation when not effectively managed.

**Conclusions:**

A range of emotions, directed at themselves and the medical school are experienced by students following exam failure. These emotions lead to a range of negative feelings and responses that affect how students make sense of and move on from the failure experience. There is a need for educators to better understand and support students to manage, reflect and contextualise their emotional responses, minimise external attribution and to enable focus on remediation and learning.

## Background

Preventing students from entering a repeated cycle of underperformance following summative assessment is a priority for medical educators due to the significant short- and long-term consequences for the student, faculty and wider society. Individuals who struggle personally and underperform academically at medical school, frequently experience problems in postgraduate training and professional practice and, therefore may present a risk to both their peers and patients [1–3]. The priority of medical educators should be to evaluate individuals who fail at summative assessment and ensure they receive the necessary support to prevent future and recurrent failure. In theory, feedback and academic support should be primary interventions after examination failure however, there is evidence that less than half of students access either of them [4]. Reasons for students not optimally engaging with feedback are multifactorial, but among those who repeatedly fail examinations, ineffective self-assessment skills [5] or resistance to change [6] appear to be present. Research into academic performance has traditionally focused on cognitive, metacognitive, and behavioural aspects of learning, demonstrating students who repeatedly fail often lack the knowledge, skills, and behaviours necessary to pass in the first place. This research has also identified that students who repeatedly fail may also lack the awareness that they are deficient in these areas [7]. However, students who repeatedly fail also experience the emotions that failure brings. Better understanding of the motivational and emotional or affective factors influencing feedback-seeking behaviour after academic failure is important for supporting individuals to positively move on from failure [8].

In the psychology literature, Boekaerts’ dual-processing model describes the relationship between emotion, motivation, metacognition, self-concept and learning [9]. The model describes two parallel processing modes: (a) a mastery or learning mode and (b) a coping or well-being mode. If students perceive a threat to their well-being, negative cognitions and emotions are triggered, and strategies are then directed to protect the ego from damage, even if that means not reading comments intended to help improve future performance [10–13]. Conversely, if or when there is no threat to well-being, students are comfortable improving their competence, triggering positive cognitions and emotions, and moving onto the mastery pathway. The challenge for medical educators is to ensure students are supported to orient themselves onto a mastery learning pathway and are helped to manage their emotions to stay there after academic failure. This challenge is significant because low-performers in examinations can inadvertently get trapped in a downward cycle of underperformance, particularly those at risk of repeated failure, due to the emotional impact of experience [6].

There are also contextual factors specific to medical education that influence levels of engagement with feedback after an academic failure, such as the burden of assessment and avoidance of receiving what may be perceived as “negative” feedback. The significant volume of summative assessment in undergraduate training can lead to students who repeatedly fail, suppressing their motivation to excel and become the best doctor possible, towards focusing more on the need to pass or get through, i.e. adoption of performance mindsets [14]. This pragmatic approach, including the adaptive change towards assessment-driven learner behaviour, can be maladaptive with evidence that students may not even engage with feedback when given too close to the next exam [15]. On occasion, non-engagement can also be driven by individuals having grief-like reactions when even just contemplating the prospect of academic failure, regardless of whether the failure is actually likely or not [16]. Given students who need feedback and support the most after academic failure are also more likely to demonstrate feedback avoidance [17] and fail to attend remediation even after agreeing to do so [16], there is a real need to investigate the personal, behavioural and environmental factors that conspire to perpetuate this conscious or unconscious behaviour. To our knowledge, whilst the emotional experience of failure has been previously explored [18], this is the first study to explore the impact this has on receiving feedback in undergraduate medicine.

The aim of this research was to explore the personal experiences of medical students receiving feedback after failing an examination in order to identify if persistent emotional reactions exist and the nature of these and if they impact cognition, motivation for learning and subsequent receptiveness to feedback.

## Methods

### Context and participants

Students on the 5-year undergraduate MBBS programme at the University of Nottingham were invited to participate in the study. The programme is separated into a pre-clinical phase, lasting two and a half years, followed by a clinical phase of the same length with an intake of greater than 300 students per year. Prospective participants had undertaken written examinations and a single, summative, Objective Structured Clinical Examination (OSCE) in each year of the first two and half years of the programme. Likewise, in the clinical phase, students undertook a written examination (comprising a combination of single best answer and extended matching questions totalling between two and four hours of testing depending on the year of the course) and a single OSCEs at three further assessment points prior to graduation. A pass in all components was required for progression to the next stage of the course. Following each assessment, students received feedback. For written assessments, feedback comprised a breakdown of the learning objectives assessed and the proportion of questions they answered correctly for each objective. For the OSCE, feedback comprised information on whether the student had passed or failed each station, including comments from the examiner about the quality of their performance from a list of pre-defined descriptors. Students who did not pass assessments (at the time of the study, typically around 5–10% of the cohort) were offered opportunities for further teaching. No formal remediation programme was provided. Changes have subsequently been made including the appointment of a remediation lead. All students in the fourth year of the course who had failed either the written examination or OSCE in the clinical phase of the programme and had subsequently passed at a resit (a requirement of the ethics committee) were invited to take part in this study. So as not to cause distress by directly emailing those who had failed assessments, all students in the cohort were contacted by email, and those with experience of exam failure who were interested in participating were asked to self-identify.

### Methodological approach

A mixed methods investigation was designed to guide the research process in this study. A qualitative investigation was necessary to explore the personal and subjective experiences of medical students receiving feedback after failing an examination. Interpretive phenomenological analysis (IPA) was chosen as the qualitative approach since IPA is particularly useful for exploring in-depth how people make sense of significant life events such as academic failure [19, 20]. A purposive sample of those who had experienced failure is ideal for IPA since representing individual perspectives and providing in-depth data about the phenomenon of receiving feedback after failure was more important than characterising the general experience of a population of students about receiving feedback. Likewise, IPA helped to explore how a relatively homogenous group of individuals, who on the one hand, encountered a common event such as receiving feedback after academic failure, experienced diversity in their responses to it [21].

Additionally, a content analysis approach was used for investigating the quality and quantity of words in the participants’ interviews, predominantly to emphasise the language dimension in the phenomenological and interpretative analysis. The content analysis approach—linguistic inquiry and word count (LIWC)—augmented the preceding qualitative investigation by analysing data in a different way by focusing attention on the specific words used to describe emotions and cognition on motivation for learning, ability to receive feedback and subsequent response to failure. LIWC is a quantitative computational linguistics method that was developed to characterise the use of language in personal narratives [22, 23] thereby allowing a deeper insight into where students were with respect to moving on from the failure, for example – “still thinking about it”, “still upset about it”, or “making adaptive change following it”. LIWC software was used to calculate the relative prominence of different linguistic dimensions—related to cognitive, emotional, and motivation processes, among others—underlying the interview transcript. The analysis focused on ‘function’ words, including pronouns, prepositions, articles, and adverbs, which in the context of this study, provided additional information into the personality and emotional state of the storyteller [24]. The LIWC analysis serves as a content analysis approach, thus making it a reproducible and stable measure of topics—as it cannot be biased by the views of the coder, and provides validity through an inherently limited evaluation of how language use maps onto underlying concepts (i.e., linguistic dimensions). Therefore, in this research, the use of LIWC was also used to provide converging evidence for the interpretative analysis of the interviews. The use of LIWC allows an external validation of the inherently subjective process of IPA conferred by its double hermeneutic. While both LIWC and IPA have different strengths, both analytic strategies share similar goals and the convergent interpretations strengthen the conclusions presented.

### Data collection and analysis

**Fig. 1:**
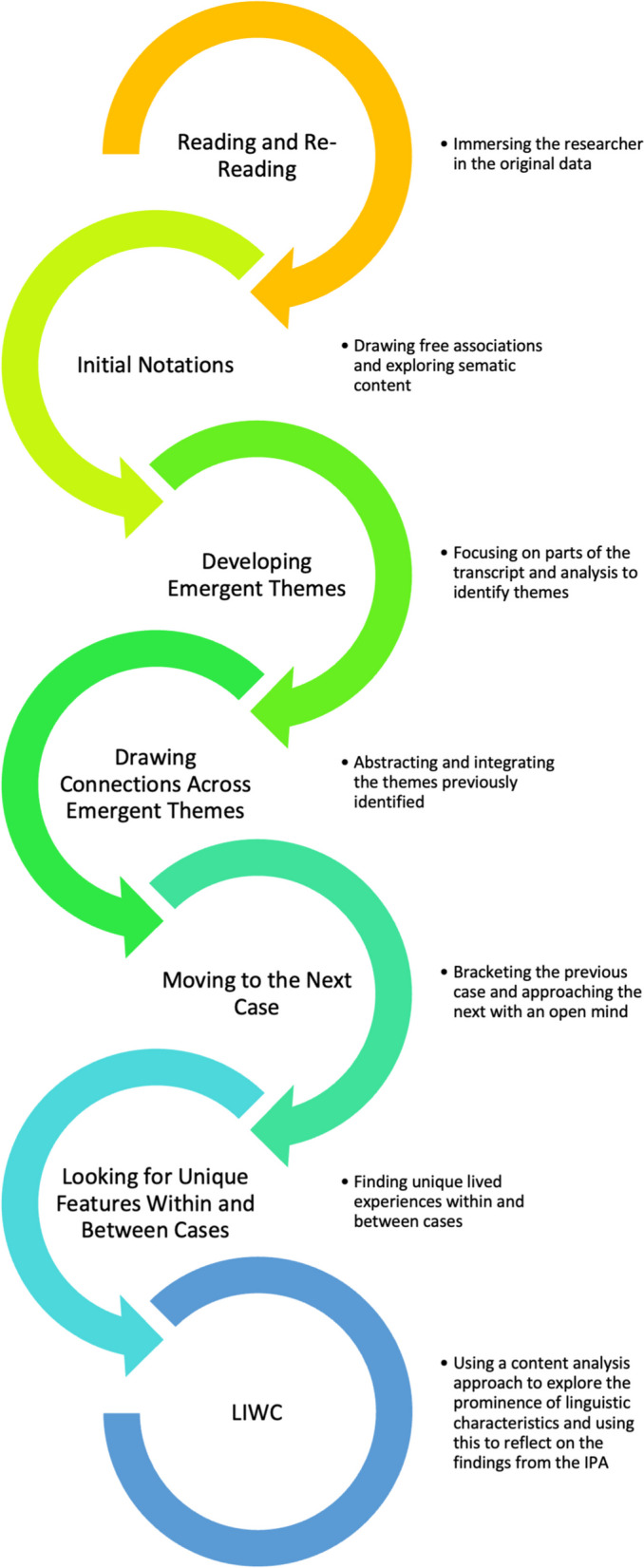
The steps of IPA data analysis supported by LIWC (adapted from Charlick, McKellar and Pincombe, 2016 [25] and Smith et al. 2009 [19])

Semi-structured interviews were used to collect data about participants’ experiences of receiving feedback twelve weeks from their initial academic failure event and approximately eight weeks following their successful reassessment. A topic guide (See Appendix) was constructed to guide the interview process and reviewed and adapted following each interview. All interviews were conducted by RJ, and the audio recording transcribed verbatim by the interviewer. To protect identity and maintain confidentiality (as per ethics approval) participants were assigned a pseudonym prior to the analysis. Given the nature of the topics discussed, participants were not invited to review and correct the transcript. Interviews were conducted via *Microsoft Teams®* as students were on placement outside the university. The idiographic nature of IPA necessitated a detailed analysis of each case involving an initial review of the data and an examination of small units of text to annotate and describe both the use of language and imagery among participants. Next interpretative coding was used to look at the transcript as a whole, identifying patterns and emergent themes present in each individual account, particularly focusing on any aspects of an idea that were unique to that case. Analysis was undertaken by hand using pen and paper. Thereafter, a cross-case analysis was conducted to identify themes shared between the cases. Finally, the LIWC content analysis was undertaken to check the consistency of linguistic dimensions as well as the themes that were uniquely extracted across participants’ transcripts, since validation studies have demonstrated that the word dictionary and categories used in LIWC account for 86% of spoken and written language [22] Fig. 1.

### Reflexivity

The interpretative element of IPA in this study centred around the interaction between the research team’s understanding of the emotions experienced by students and how the students described by their sense-making process after failing at assessment [26]. RJ and RP are clinicians and medical educators with experience supporting students following examination failure and researching this topic. RJ and RP have personal experience of failure during their medical education. PH is a senior medical educator and has extensive experience supporting students through academic and personal difficulties as well as in medical education research. CRM is a psychology researcher with expertise studying emotion. Neither RJ nor RP had any direct involvement with the remediation of these students but may have encountered them at other points during the course. PH would likely have provided pastoral support during the participants' university career, however, was intentionally kept unaware of the participants' identity to avoid any bias. After the reading of the interview transcripts, RJ and PH reflected on, and made explicit their own assumptions about their interpretation of the narratives challenging each other as appropriate. RP interrogated the interpretation before CRM provided further scrutiny following the LIWC.

Given mixed methods research involves integration of assumptions from different paradigms (participant's objective and subjective perceptions), four types of triangulation were necessary: i) methodological triangulation, with the use of more than one data collection technique (semi-structured interviews and LIWC analysis); ii) data triangulation, with the use of multiple data sources (text and numbers); iii) investigator triangulation using all members of the research team; and (iv) theoretical triangulation (dual-processing model). Reflecting over the extent to which this procedure was followed, ensured presuppositions (biases) did not impact unknowingly on the process of analysis and interpretation wherever possible.

### Ethics

The University of Nottingham Faculty of Medicine and Health Science Ethics Committee granted approval for the study. Informed consent was taken from all participants prior to participation in the project. All methods were carried out in accordance with relevant guidelines and regulations.

## Results

Five participants volunteered to take part in the study however one individual chose to withdraw before interview for personal reasons. Of the four remaining participants, three individuals were female and one male. All participants experienced failure in their third-year exams, i.e. in the first of the clinical phase of the course. All participants had failed their written examinations at this progression point and additionally, two had failed the (OSCE). The four semi-structured interviews ranged from forty-five to seventy-five minutes. All participants experienced multiple emotions after failing assessments. The strength and type of emotion varied across the different participants and impacted on their relationship with both the university and their approach to feedback. These ideas are illustrated as follows:

- 1) Emotions including anger, fear, anxiety and sadness

Nik experienced shock and anxiety, which triggered other emotions such as feeling inadequate and inferior.

*“I think those are probably the worst days of my life (referring to the period immediately after finding out they had failed the assessment) the panic, stress, I don’t even know how I got through those days, to be honest with you... I won’t lie to you, there was a lot of crying. I’m worrying whether I’m actually going to be able to pass this; what don’t I understand? Where am I going to start preparing this? [The resit exam]”*

Nik found transitioning to university quite difficult because previously they were a high-flier and were now experiencing failure for the first time, further exacerbating feelings of inadequacy and worthlessness.

*“Initially, I was rejected with my university application, but later got in, so you start thinking, are they going to realise they made a mistake and just get rid of me? You are kind of top of your class at school, and then you get to med school, and you realise you’re not all that. Your kind of, just scraping it. You’re asking yourself; do I deserve to be here or not?”.*

There was also anxiety and shame for Nik borne out from the consequences following failure financially but also the potential stigma for them personally.

*“The fear was of repeating the whole year again (if they did not pass their reassessment), partly that’s the student fees that you are paying again, but also having no friends because you're not familiar with the year below.”*

*“The shame once people kind of recognise that they’re not familiar to you (if they were required to repeat the year). And people asking like, well, where are they? Oh, they failed and are in the year below now.”*

This was Jo’s first experience of academic failure, and they felt a significant amount of anger—both towards themselves and the medical school.

*“I didn’t expect to fail this kind of thing and I was super emotional for a while. I was so angry at myself for being stupid, I guess. The feeling of being a failure, the anger at the university and then the anger at myself “*

Jo was fearful of the repercussions on their future career, and the fear drove further feelings of anxiety.

*“I was scared, of course, because I failed, and then scared by how much it would affect my grades in the future. It could have such an impact on my entire life, which I have worked so hard for. It just made the anxiety so much worse”.*

The anxiety manifest itself as Jo losing their appetite and having difficulty sleeping.

*“I didn’t eat, didn’t sleep, because I had nightmares about failing, I was scared to sleep because of it.”*

Shim had pre-existing mental health problems, and failure triggered a depressive episode, including thoughts of self-harm.

“I think those were the worst days of my life (again referring to the period after finding out they had failed the assessment). It was just awful, I felt absolutely horrible … It’s hard to physically do anything when you are in that sort of headspace, where your just exhausted all the time, and you just want to stay in bed … I really wanted to kill myself.” *^1^

Ki described the emotional experience as a type of trauma.

“There is no package of care for people who are re-sitting, no one acknowledges the trauma, it’s such a difficult period in life.” (NB: At the time all students were given the details for support and wellbeing services when informed that they had been unsuccessful and a personalised support meeting offered however this student suggests they had not been aware of this or had not felt able to access it).

For Ki, the sense of shame was the worst feeling of all.

*“And then I think, everyone knows that ’I’d failed. I can’t not admit I find that embarrassing, I feel quite stupid because of it (failing)… At the time, it just felt like the worst-case scenario, and with all the weight of failure and embarrassment, that other people could see and then maybe(they) think you don’t know enough.”*

However, there was also anger, and they felt themselves having a short temperament as well.

*“You are just so upset that you can’t do anything, you can’t function, I was on a really short fuse”*

These emotions also prevented further engagement with accessing feedback.

*“At the time, you don’t want to know the reason why you failed (referring to receiving feedback) because it will make you feel worse. It will just make you feel bad.”*

- 2) Suspicion, mistrust and distrust

Nik’s shock and concerns about the assessment process led to suspicion about the medical school’s intentions.

*“Will they (the medical school) give you the right equipment to perform in an exam or are you going to have to use something different to make it harder for you? (referring to an examination where a piece of equipment was different, in different trusts that work with the university) In the questions, they (the medical school) use these statements to trap you, to make you think the opposite, to work out why something is not a feature of a condition.”*

The suspicion fuelled mistrust in the support offered by the medical school.

*“It (the support offered) just made me feel hopeless because I don’t know how much I can rely on what they (the medical school) are giving me.”*

For Jo, the anger catalysed a change from mistrust (i.e. scepticism about the trustworthiness of another) to distrust (i.e. a settled belief of untrustworthiness, often based on evidence or experience) because they felt the medical school did not act the way they wanted on their requests for help and support with some difficulties in the run-up to assessments, further fanning their feelings of anger when the failure occurred.

*“I told them about all the issues and they just brushed me off and then it's like look, you failed, oops, take the failing grade congratulations, and I was just so angry because of that.”*

The distrust extended beyond the medical school to the whole University and forced Jo into feeling they had to document everything just to prove their side of things in case they failed further assessment in the future.

“*The whole university in general I don’t really trust them anymore, so even if they say something is ok, I have to chase it down, I have to get proof, like email proof. I just can’t trust them anymore.” (A feeling or perception that a previous request for help was not met lead to a desire to evidence all instructions and communications with the university in case of a future dispute)*

Shim’s experience led to a mistrust of the assessment procedure, specifically the role of the examiner.

*“Everything is subjective to the examiner; the examiners are open to human error. It’s all a bit questionable really, essentially, you are just at the mercy of the examiner. No matter how hard you try, you just fail for some reason you don’t know, and it makes it all seem like it’s impossible, like they don’t want you to pass the course.”*

Shim also distrusted the feedback process due to a dissonance between their perception of what happened at the assessment and the examiner's judgement about the performance on the day.

*“There was one situation when it said in the written feedback that I didn’t do something that I did, so it just makes the whole thing more questionable.”*

A degree of mistrust and distrust was fuelled by emotions and experiences of abandonment.

*“There is no package of care, no acknowledgement, you’re sort of thrown on your own, and it’s not the best time to be on your own … There’s not enough checking in to see if we are ok, it’s kind of like you’re thrown on your own.”*

Ki’s anger manifested in suspicion and distancing themselves from the medical school.

*“The OSCEs it’s just a tick box exercise, you just have to do certain things and at the end of the day, as a doctor it’s not about ticking boxes. After you come out of that environment (the OSCE), you can just go back to normal. “*

There was a dissonance again between how Ki perceived they had done, the marking of the performance by the examiner and the assessment outcome.

*“I had, like, 90, 90, 90s in sections, but then we were really confused about how the mark came out like it did, because I don’t know where I was missing marks. It’s off, like, a tick box format, so what have I got wrong to lose 40% of the marks? I’m still confused, and we all felt.(the student body) like this (this refers to the difference between raw marks and standard set marks in written assessments).”*

Despite automatic feedback in written assessments, released with results there was also frustration that students felt they had to ask for feedback rather than receiving it automatically.

*“We always have to ask for it, it's like, yeah you can get feedback if you want, but they don’t provide it to us unless we ask. It's silly because the information is there why don’t you just provide it!*

- 3) Experiences and expectations of feedback

Nik’s expectation of feedback was that it should offer more praise rather than identify areas for improvement.

*“Telling me that, for example, I approached the bed from the wrong side, or telling me that my cannula was put in wrong, doesn’t help me. It’s good feedback when they don’t interrupt you or break your confidence, praise you on what you did well and then go through the little things.”*

Receiving feedback also caused some dissonance between Nik’s self-assessment of their ability and their actual measured performance at assessment which in turn dented self-confidence for moving forward.

*“I’m looking at the feedback and I’m like, that really doesn’t add up because I think I knew this really well and I did some of the worst on it. It really shakes your confidence, and means you don’t know what you need to revise”*

Nik possessed a strong sense of wanting feedback to avoid looking like a failure in front of others.

*“You are revising because you want to pass but also with the fear of dropping down to the year below.”*

However, there was also a sense for Nik, that success at assessment was more about luck than anything else related to study or learning.

*“There is an element of luck to it because every student can’t know every topic. So sometimes you just have to guess, and if you’re lucky, you guess right on the stuff you didn’t know.”*

Jo wanted feedback that would identify areas for improvement, and on any aspect of performance, whether big or small.

*“It’s supposed to let you know what you can improve on. Even the small mistakes, they need to point it out, that’s how you develop. I’m just like trying to find out the best way to pass, that’s it that’s what’s important.”*

Jo’s suspicion also led to the belief that staff were engaging in malevolent behaviour towards them.

*“If you hate someone, then you’re just going to give them bad feedback, like all the critical things.”*

Jo also dismissed feedback that was contrary to their self-perception about their own ability.

*“If I’m not sure about some feedback, I will go and check it out myself, then maybe disregard it if I think it's wrong*.”

Shim’s expectation about the purpose of feedback also borne out of a belief that it should be all about receiving any information to avoid failure.

*“We need to understand how our performance would be interpreted by the examiner, helping you understand what the examiner is looking for, what you need to show them. When we have teaching, there should be more emphasis on the examiner's perspective of actions.”*

Similarly, this belief also motivated them to do anything that avoided failure rather than enabled them to become the best doctor they could be.

*“My motivation is a combination of determination and fear, I don’t want to fail an exam, I can’t go through all of this again.”*

In contrast, Ki’s expectation about feedback was centred around receiving information that enabled them to be the best they could be rather than something the medical school could tick off as something they’d done because they had to.

*“We need more pointers on things that we could improve on individually rather than just the tick box stuff.”*

Ki’s description of good feedback drew from their experience of receiving sports coaching and one-to-one support in order to improve performance.

*“Its {good Feedback] where there is an individual getting to know me, knowing my strengths and weakness and using their expertise to tell me what they observe and how that would help me.”*

Ki was particularly sensitive to the perceptions of others and this judgment affected self-confidence amongst other things.

*“When you’re in scenarios where you’re not with the nicest person, who doesn’t really like medical students, so they are just really harsh to you, because of that, you know they just keep correcting you and interrupting you. It makes you really nervous, and it just breaks you down.”*

Nevertheless, ultimately, Ki was driven by the desire to avoid failure first and foremost and sought feedback or opportunities that helped them achieve this goal.

*“The failure is pushing me a fair bit, just not to replicate it because it was too stressful the first time. So, I know I need to cover the core topics and focus on them just to give me the best chance of getting an efficient grade“*

### Linguistic Inquiry and Word Count (LIWC)

Results from the LIWC content analysis, providing an objective comparison with our IPA results and are presented in Table 1. Consistent in part with the reflective nature of the interview, words related to cognitive processes were particularly prominent in the responses from all four individuals, ranging from 16.3% to 18.0%, 1. Cognitive processes included thinking-related concepts, such as use of the words ‘determine’ or ‘should’ suggesting participants had thought through the experience and identified actions required as a consequence of the failure. Words associated with affective processes were present, ranging from 5.1% to 7.2%. and consistent with the IPA, were predominantly negatively orientated, with anger and sadness commonly featured. Discussion of motivational drivers, including achievement and power, were also identified in both approaches. Example words and relative use of each of these are included in Table 1.

**Table 1:** Percentages of relevant linguistic dimensions for each interview from the LIWC analysis

Dimension	Sub Dimension	Example Words	Study Participants			
			Nik	Ki	Jo	Shim
Cognitive Processes		determine, should	16.3%	16.3%	16.4%	18.0%
	Insight	decide, explain	5.4%	5.0%	3.2%	4.6%
Relativity		deeper, recently	10.0%	10.7%	8.1%	8.7%
Affective Processes		hope, stupid	5.1%	5.3%	7.2%	5.4%
	Negative Affect	panic, worst	1.4%	1.9%	2.9%	1.5%
	Anger	angry, screwed	0.2%	0.2%	0.5%	0.1%
	Sadness	crying, depressed	0.4%	0.6%	1.1%	0.4%
Motivational Drives		love, practice	5.3%	5.5%	6.6%	6.3%
	Achievement	able, plan	1.4%	1.9%	2.1%	1.9%
	Power	help, weakness	1.6%	1.7%	2.0%	1.9%
Unique Words			(none)	(none)	swear	death
**Total Linguistic dimension coverage**			87.5%	92.5%	93.8%	89.7%

Variability in dimension values, i.e., prominence of different language concepts, did converge with findings identified through IPA. Shim’s preoccupation with distrust contributed to the greater use of words weighted on cognitive processes. Jo’s negative emotions and feelings of anger was reflected in greater use of words associated with the linguistic dimensions of the LIWC content analysis. Nik and Ki demonstrated more use of insight and relativity, showing more understanding of the overall situation and context, in comparison to both Jo and Shim. Some dimensions were only present in some participants’ transcripts, as noted as ‘unique’. ‘Death’ was present, convergent with the quote related to self-harm from Shim. The malevolent behaviour that Jo suspected was detected through the use of swear-related terms (“they screwed us over”).

Linguistic Inquiry and Word Count (LIWC) Summary.

## Discussion

This research discovered that a range of emotions was experienced by these medical students after failing summative assessments, influencing both their feedback-seeking behaviours and their motivation for learning. The findings identified emotions affected both the way these students thought about and made sense of failure, and how they attributed reasons for their failure, resulting in feelings of mistrust or distrust of the medical school. The study identified that these students were not seeking feedback intended to help them study more effectively over the longer term, but instead clear instructions for the short-term and how to pass next time. The findings from this research offer new and novel targets for further research into the role of emotions following failure at assessment, but also the way medical educators support students after failure, particularly around emotional support, regaining trust and giving feedback.

Emotions included shock, fear, anxiety and anger, through to sadness and disgust, all of which are described as universally experienced by the participants [27, 28]. Experiencing these emotions following failure is not necessarily a negative outcome for individuals with a mastery orientation [29], since having a sense of guilt can be a useful acknowledgement of something going wrong and a need to learn lessons from the experience. However, the findings from this research identified that when emotions led to feelings of shame or embarrassment, students’ cognition and behaviours were maladaptive and self-sabotaging (i.e. adopting ineffective but comforting strategies as negative thoughts prevent a growth mindset) in terms of responding to failure and receiving feedback. Shame is a deep-seated feeling and can be destructive for responding to failure because it corrodes self-efficacy, including any beliefs about capability to change [30, 31]. One type of shame is where individuals feel embarrassed because of their behaviour, and the other is when individuals feel that others are embarrassed by them. This second type of shame emotion appeared to have been experienced by participants in this research and contributed to a feeling that the medical school or the faculty wanted to “get rid” of them so as not to embarrass the school It has been suggested, in a clinical context, that a fear of “bad” feedback was a barrier to accessing it, and was associated with a fear of seeking feedback which may “shine a light” on their performance [32]. Whilst no individuals were subject to any direct acts of shaming, the very outcome of failure was enough to trigger shame, embarrassment, and a sense of humiliation, producing a fear of similar future experiences. Although educators may not be able to prevent individuals from experiencing such feelings, the focus of support could perhaps be on acknowledging and managing this emotion whilst also enabling individuals to develop effective emotional regulation.

Emotional regulation is necessary for effective performance whether on an academic task [9] high-performance sports event [33] or high-pressure emergency situation [34]. Likewise, emotional regulation is core to effective adjustment following failure [35] and likely to matter more for students who are orientated to protecting their well-being or self-worth at all costs. However, remediation interventions often focus on providing “more of the same” teaching as before [36], rather than strategies that help students manage themselves or their emotions around the time of, or during the assessment. Insights from sports psychology could be of help in this regard, especially focusing on supporting students to better manage their emotions around significant high-performance events such as when preparing for, undertaking, or moving on from the assessment [37].

Adjustment following summative examination also requires effective self-regulation, and particularly with aspects related to appropriate attribution of the reasons for success or failure. Attribution theory is well described, with some individuals known to prefer externally associating reasons for failure [38]. This research suggests the emotions experienced in and around the failure, may have contributed to the making of those judgments with feelings running high, and likely influencing the negative reaction toward the medical school. In some cases, there were feelings of suspicion linked to anger that led to distrust, whereas in others, feelings of avoidance and aversion linked to disgust led to mistrust and a breakdown of relationship with the medical school. Previous research has identified trust in the medical school as lacking among students [6], further highlighting another reason why creating a safe learning environment is so important in a medical education context, particularly for those more orientated to protecting their well-being or self-worth.

As a consequence of the emotions and feelings experienced by students after failure, and in some cases what sounded like grief-like reactions, individuals’ receptivity to, and readiness to receive feedback was minimal. Even though there is evidence that feedback or remediation aimed at helping students get through the resit or the next exam is ineffective for preventing future failure [39], students still seek out this type of information during remediation. This research also suggests a performance mindset may not only exists before assessment, but even after failure, and the likelihood of educators changing it when students behave in this way may be low. Despite feeding back in these situations feeling uncomfortable for educators, there is still a moral responsibility to provide it regardless of whether students are ready to receive it in a way that it will have some utility [40]. What remains unclear from this research is whether a performance mindset was preferred as a temporary frame due to the failure result, and a pragmatic need to just get through the exam in order to remain on the course [29]. A longitudinal exploration of student’s performance mindset may provide further information.

There are a number of strengths and limitations to this study. This is the first IPA study described within medical education to use a complementary method of data analysis to increase the depth of analysis, but also increase the trustworthiness of the findings. Most IPA studies rely on the interpretation of researchers alone, whereas this study also used software analysis alongside to strengthen the triangulation process and thereby increase the dependability of the data categorisation. Although the participant narratives were vivid and offered richness into the experience of failure, the focus of the interview was on one summative assessment only. Although similarities in emotions expressed and feelings experienced may exist among participants after failure, the stories are likely to be even more diverse across different assessments across the course. Another limitation is that the interview schedule permitted limited exploration of their lived experience, whereas remediators will instinctively know that exploring their whole stories is often necessary for practical purposes – re-engaging them, regaining their trust, and restoring their confidence in the system. The use of LIWC as a complementary approach helped quantify the relative use of different linguistic dimensions, as a form of objective content analysis. The utility of this approach would also be strengthened by having a second interview further after the aftermath of failure, to explore how emotion and motivation had changed over time.

Finally, one of the features of IPA studies is the interaction between the researcher’s understanding of the phenomenon under investigation and the participant’s perceptions of the sense-making process [26]. This research was conducted by an academic team researching the very students that they also had responsibility over, albeit indirectly, in an educational context. The ethical issues related to this proximity of the distance between the researcher and the participant were considered as part of the approval process, however the interpretation of the findings from the research may have been influenced nonetheless. The complementary LIWC analysis demonstrates sufficient distance was retained between the researcher and the participant, and the LIWC also provides another way of triangulating the researcher’s interpretation of the phenomenon under investigation and the participants’ self-perceptions of it.

## Conclusions

This research has identified that medical students experience a range of emotions following failure in summative assessments, and the direct impact of these include emotions affecting both the way that they make sense of the failure experience, as well as the way individuals perceive and interact with the medical school and university. These emotions can also have a detrimental impact on their desire to seek and engage with feedback, along with their motivation for learning in the future. Medical schools should consider supporting students to better manage their emotions after failing experiences otherwise interventions such as feedback or remediation may not be as effective as they could or should be. Research exploring the impact of such emotional regulation and management strategies is likely to be of wider benefit to the medical education community.

## Data Availability

The datasets generated and/or analysed during the current study are not publicly available due to the sensitive nature of the student's experiences but are available from the corresponding author on reasonable request.

## Appendix

### Interview Topic Guide

How would you describe feedback, what do you feel it is? Do you have any examples of what feedback is or isn’t?

What does good feedback look like to you? Can you describe any particularly good or bad experiences you have had?

Is there an occasion when you have received feedback at university where it has been either particularly helpful or unhelpful?

What do you remember of the days after you found out you had failed an exam?

What do you remember of the feedback after the exam?

What did you do with the feedback, did you change anything in the way you work?

## Abbreviations

IPA: Interpretative phenomenological analysis
LIWC: Linguistic inquiry and word count
OSCE: Objective structured clinical examination

## Footnotes

1 ˙* At this point the interview was paused and the student confirmed they did not need additional support at this time and that they were happy to continue.
